# Preparation and Performance Improvement Mechanism Investigation of High-Performance Cementitious Grout Material for Semi-Flexible Pavement

**DOI:** 10.3390/polym15122631

**Published:** 2023-06-09

**Authors:** Peifeng Cheng, Guangtao Ma, Yiming Li

**Affiliations:** 1 School of Civil Engineering, Northeast Forestry University, Harbin 150000, China; chengpeifeng@nefu.edu.cn (P.C.); maguangtao1122@163.com (G.M.); 2 Longjian Road and Bridge Co., Ltd., Harbin 150001, China

**Keywords:** cationic emulsified asphalt, principal component analysis, road performance, scanning electron microscopy, semi-flexible pavement material

## Abstract

Semi-flexible pavement material (SFPM) combines the advantages and avoids the disadvantages of asphalt concrete flexible pavement and cement concrete rigid pavement. However, due to the problem of interfacial strength of composite materials, SFPM is prone to cracking diseases, which limits the further application of SFPM. Hence, it is necessary to optimize the composition design of SFPM and improve its road performance. In this study, the effects of cationic emulsified asphalt, silane coupling agent and styrene–butadiene latex on the improvement of SFPM performance were compared and analyzed. The influence of modifier dosage and preparation parameters on the road performance of SFPM was investigated by an orthogonal experimental design combined with principal component analysis (PCA). The best modifier and the corresponding preparation process were selected. On this basis, the mechanism of SFPM road performance improvement was further analyzed by scanning electron microscopy (SEM) and Energy Dispersive Spectroscopy (EDS) spectral analysis. The results show that adding modifiers can significantly enhance the road performance of SFPM. Compared to silane coupling agents and styrene–butadiene latex, cationic emulsified asphalt changes the internal structure of cement-based grouting material and increases the interfacial modulus of SFPM by 242%, allowing cationic emulsified asphalt-SFPM (C-SFPM) to exhibit better road performance. According to the results of the principal component analysis, C-SFPM has the best overall performance compared to other SFPMs. Therefore, cationic emulsified asphalt is the most effective modifier for SFPM. The optimal amount of cationic emulsified asphalt is 5%, and the best preparation process involves vibration at a frequency of 60 Hz for 10 min and 28 days of maintenance. The study provides a method and basis for improving the road performance of SFPM and a reference for designing the material composition of SFPM mixes.

## 1. Introduction

Semi-flexible pavement material (SFPM) is a rigid, flexible pavement material formed by infusing cement-based grout into a large void asphalt mixture [1]. It combines the advantages of asphalt concrete flexible pavement and cement concrete rigid pavement. From the perspective of material strength formation, asphalt concrete pavement forms material strength mainly through interlocking between aggregates, while infilled semi-flexible pavement forms material strength through the intercalation of asphalt mixture aggregates and cement-based grouting materials [2,3]. Therefore, SFPM has higher strength and stability than dense-graded asphalt mixtures and better resistance to deformation, water damage, and slip than porous friction course (PFC) [4,5]. Therefore, SFPM is often used in special roadways, such as heavy-duty pavement, intersections, and bus stops [6,7].

The first semi-flexible pavement originated in France, and the first trial pavement of semi-flexible pavement was carried out on the runway of Cognac Airport in France in 1954 [8]. Subsequently, the performance of semi-flexible pavement was studied in depth in the UK and the US. The study showed that semi-flexible pavement can improve high-temperature stability and extend the service life of the pavement [9,10]. At this stage, the research on SFPM road performance mainly focuses on the improved design of semi-flexible pavement structures and the selection of new grouting materials [11].

Researchers have conducted much research on the structural composition of SFPM. Ding et al. [12] constructed a finite element model of SFPM using digital image processing techniques, characterized the internal conditions of SFPM using the three-phase material structure, and analyzed the weak points of SFPM cracking and damage. They pointed out that SFPM has the highest probability of cracking at the interface of asphalt and grout materials. Zhou [13] analyzed the damage pattern of semi-flexible pavement. The results showed that damage to semi-flexible pavement usually occurs at the cement–asphalt interface and the cement itself. Yang et al. [14] investigated the fatigue performance of SFPM under repeated loading. It was found that the pore volume of the asphalt mixture skeleton significantly affects the durability of SFPM. Lei Cheng [15] utilized the uniform design method to create a ratio for cement mortar. Based on the optimal ratio, he introduced the volumetric method of asphalt concrete to determine the gradation of the matrix asphalt mixture. Cheng proposed a ratio design method for semi-flexible pavement materials [16]. Husain et al. [17] utilized a two-parameter analysis method to investigate the correlation between the gradation types of matrix asphalt mixture and the mechanical properties, fatigue properties, and volume parameters of the semi-flexible pavement material. The study also examined design parameter indicators. Chen et al. [18] used the discrete element method to analyze the effect of the compaction method on the SFPM volume parameters. The results showed that the compaction method significantly affected the vertical and horizontal pore volume distribution of SFPM, and the pore volume distribution of the specimens obtained by rotary compaction was more uniform than that by vibratory compaction. With the advancement of technology, researchers [12,18,19] have started utilizing digital image processing techniques in combination with finite elements to create a digital model of the structural composition of SFPM. They use software such as ANSYS to analyze the mechanical response of semi-flexible pavements and simulate the performance of the base asphalt mixture used in semi-flexible pavement materials.

Interfacial editing theory has gained significant attention in recent years, particularly in the field of composite materials. Studies have demonstrated a clear relationship between the mechanical properties of composites and the interfacial compatibility and strength within the material. The addition of interfacial modifiers to composites has been found to enhance both compatibility and strength, leading to improved overall performance of composites [20]. A study has shown that the interface between cement and asphalt composites does impact the performance of semi-flexible pavement materials. The study compared the microscopic characteristics of the asphalt mortar interface and the macroscopic road performance of asphalt mortar before and after interface optimization. Additionally, the study revealed the positive effects of interface modifiers on semi-flexible pavement materials [21].

As a composite material, SFPM consists of a matrix asphalt mixture as a skeleton and cementitious grouting material to form a skeleton-fill structure. According to previous research [22], the mechanical properties of cementitious grouting materials play a crucial role in determining the road performance of semi-flexible pavements. Previous research has demonstrated that the semi-flexible pavement material’s strength contribution from the cement-based grouting material is greater than that of the matrix asphalt mixture. Additionally, the uniaxial compressive strength of the semi-flexible pavement material is comparable to that of the cement-based grouting material. This means that the strength of the semi-flexible paving material is primarily determined by the strength of the cement-based grouting material [11,23,24]. Cai [25] introduced the acoustic emission technique and suggested that cement grouting can significantly reduce the damage strain of the material by examining the damage process of SFPM and its porous asphalt mixture in the uniaxial compression test, the results. A. Setyawan [26] concluded that the strength of SFPM depends mainly on the strength of the cement binder, and the compressive strength of cold-mix grouted composites is lower than that of hot-mix grouted materials. Yang et al. [27] showed that the addition of waste rubber powder can improve the low-temperature crack resistance of SFPM, and the improvement is related to the injection method. Wang et al. [28] improved the performance of SFPM by incorporating different flexible latex materials into cement-based grouting materials. The results suggested that the performance of SFPM was significantly improved by adding polymers. Liu [29] analyzed the effect of phase-change materials on the road performance of SFPM. The results showed that phase-change materials significantly improved the low-temperature crack resistance of SFPM but decreased the high-temperature stability and water stability of SFPM.

In summary, the strength and structure of SFPM are greatly influenced by cement-based grouting material. The road performance of SFPM can be improved by enhancing the performance of cement-based grouting material. The use of SFPM in carrying heavy traffic is limited by its trend to crack due to the rigidity of cement-based grouting material and structural limitations of the composite material. To fully utilize the benefits of SFPM in heavy traffic applications, it is imperative to address its low-temperature performance deficiency as an urgent issue. Therefore, the study compared and analyzed the improvement effect of modifier types and dosage on the performance of SFPM. The SFPM preparation process parameters with good overall performance were selected using principal component analysis (PCA). In addition, the mechanism of modifiers for improving the road performance of SFPM was investigated by scanning electron microscopy (SEM) and EDS spectral analysis.

## 2. Materials and Methods

### 2.1. Materials

#### 2.1.1. Asphalt

SBS (styrene–butadiene–styrene) modified asphalt was selected for testing, provided by Yuxuan Waterproof Material Co., Ltd., Shouguang, Shandong province. Its basic physical properties are shown in Table 1.

#### 2.1.2. Cement

The test was conducted using ordinary 42.5 silicate cement, and the results met the requirements according to the testing of each technical indicator. The specific test results are shown in Table 2.

#### 2.1.3. Modifiers

The modifiers used in the study were silane coupling agent, cationic emulsified asphalt, and styrene–butadiene latex.

**Figure 1:**
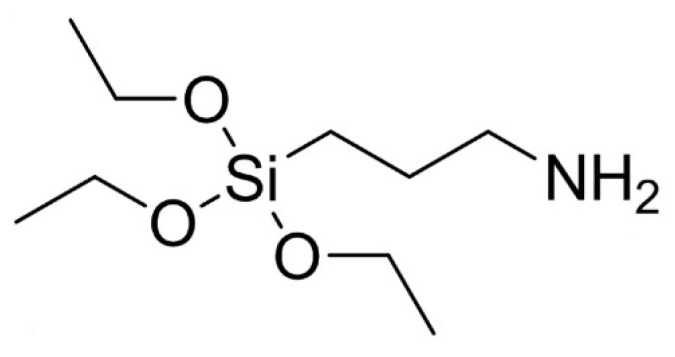
Molecular structure of the KH-550-type silane coupling agent.

The silane coupling agent is a special substance that can chemically react with both organic and inorganic materials. The agent is a polar group of pro-inorganic molecules at one end and an amino-functional silane of pro-composite materials at the other end. It can form a chemical bond with the composite material to improve its interfacial strength and performance [30]. The KH-550-type silane coupling agent was used in the test, whose chemical composition is r-aminopropyltriethoxysilane. Its molecular structure diagram is shown in Figure 1, and the specific indicators are shown in Table 3.

Cationic emulsified asphalt is a special material specially processed by a variety of surfactants, cationic shale inhibitors, and a certain range of softening points of asphalt. The cationic emulsified asphalt was provided by Rain Shield Waterproofing Technology Co., Ltd., Weifang, Shandong province and tested according to AASHTO M140. The specific indicators are shown in Table 4.

Styrene–butadiene latex is a stable emulsion made of butadiene and styrene by low-temperature polymerization, which is a creamy white aqueous dispersion with a blue-purple luster. The butadiene latex used in the test was provided by Jitian Chemical Co., Ltd., Shenzhen. The specific indexes are shown in Table 5.

### 2.2. Methods

#### 2.2.1. Design of the Base Asphalt Mixture Ratio

The test aggregate gradation was adopted as SFAC-13. Three aggregate gradations were trialed by varying the passage rate of 2.36 sieve pores, i.e., the median value of the pore passage rate of a 2.36 sieve with ±3%. The final aggregate gradation was determined and shown in Table 6. The connected void ratio of the matrix asphalt mix at this aggregate gradation is 23% (±0.1).

The surface area (*A*) of the aggregate was calculated according to Equation (1) for the median grade S-2. Based on the desired asphalt film thickness (*h*), the initial asphalt dosage of the mix was calculated according to Equation (2) $P_{b}$. In this study, the asphalt film thickness *h* of the matrix asphalt mixture was chosen to be 10 μm.

(1) $$A=\frac{2+0.02a+0.04b+0.08c+0.14d+0.3e+0.6f+1.6g}{48.74}$$

(2) $$P_{b}=hA$$

where:

*A* is the total surface area of the aggregate;

*h* is the asphalt film thickness;

*a*, *b*, *c*, *d*, *e*, *f*, and *g* denote 4.75 mm, 2.36 mm, 1.18 mm, 0.6 mm, 0.3 mm, 0.15 mm, and 0.075 mm sieve passage rates (%), respectively.

**Figure 2:**
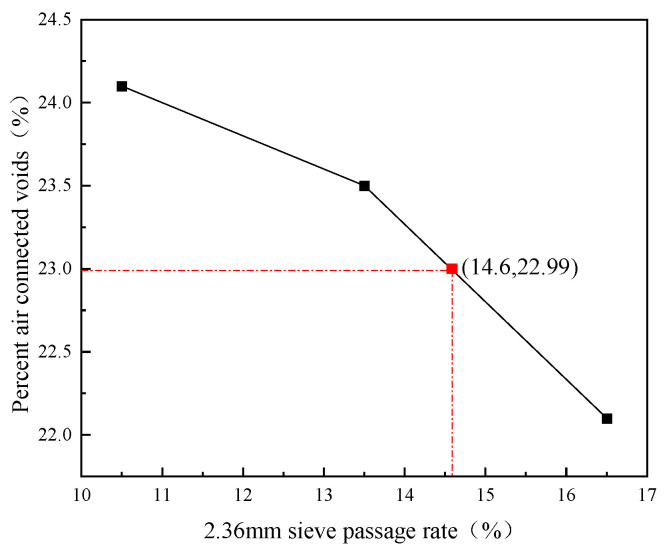
The relationship between the 2.36 mm sieve passage rate and the percent air connected voids.

The relationship curve between the 2.36 mm sieve passage rate and the percentage of connected air voids was obtained, as shown in Figure 2. The base mix aggregate gradation corresponding to the target percent connected air voids of 23% (±0.1) was selected, with a final determination of a 2.36 mm sieve passage rate of 14.6%, an expected percent air connected void of 22.99%, and a gradation of S-4.

**Figure 3:**
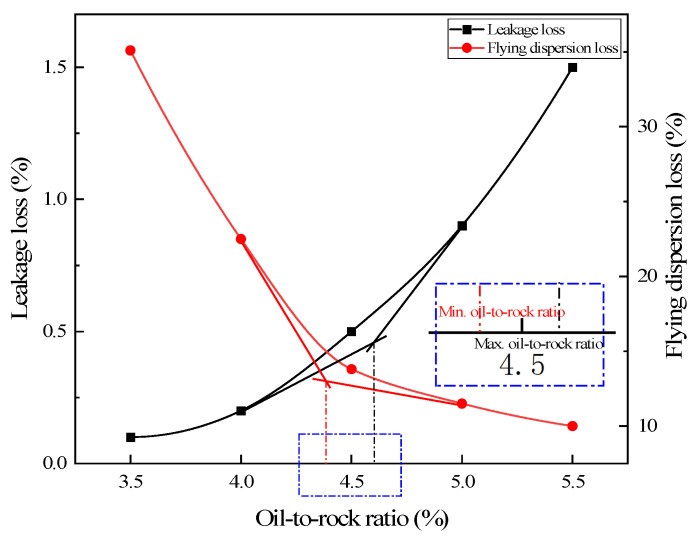
Results of Schellenberg test and Cabtabro test.

The amount of asphalt corresponding to the S-4 gradation was calculated according to Equations (1) and (2), and the Schellenberg test and Cabtabro test were performed. The test results are shown in Figure 3.

As shown in Figure 3, the slope of the curve appears to change significantly at about 4.5%. The tangents are made from the left and right sides of the curve, and the resulting intersection point is the maximum oil-to-stone ratio, which is 4.6%. As shown in Figure 3, the slope of the curve at 4.5% changes significantly. The tangents are made from the left and right sides of the curve, and the intersection point is the minimum oil-to-stone ratio, which is 4.4%. Therefore, the best oil-to-stone ratio is within 4.4–4.6%, and 4.5% is taken as the best oil-to-stone ratio.

**Figure 4:**
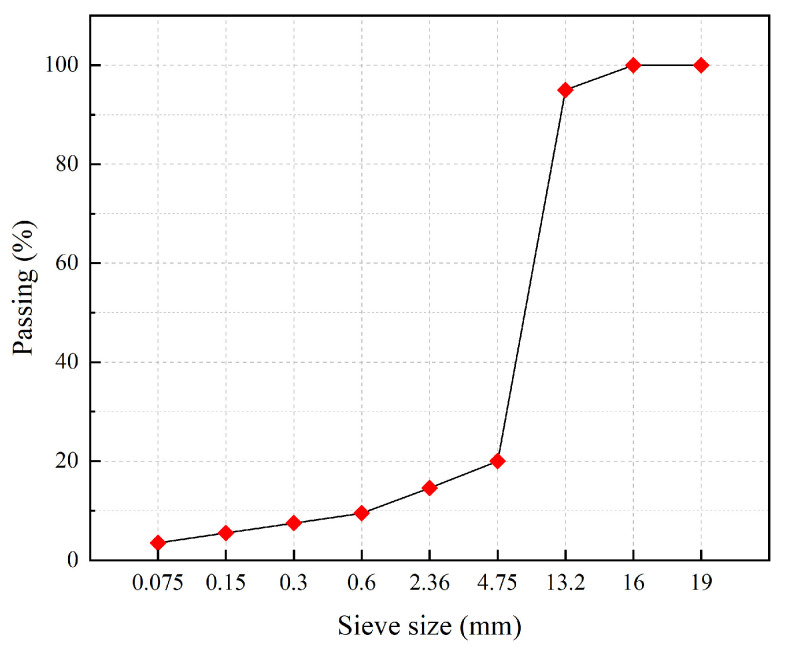
S-4 gradation.

The final test-grade S-4 composition is shown in Figure 4. The aggregates used are divided into coarse aggregate, fine aggregate, and mineral powder according to the particle size. Among them, basalt is used for coarse and fine aggregates, and high-quality limestone mineral powder is used for mineral powder.

The best oil-to-rock ratio of the matrix mixture was determined to be 4.5% by the Schellenberg precipitation leakage and Kentucky Fortress dispersion test. Its main technical performance indicators meet the specification requirements, and the specific test results are shown in Table 7.

#### 2.2.2. Design of the Cement-Based Grouting Material Ratio and Determination of the Grouting Volume

The water–cement ratio was finally determined to be 0.5 by changing it and taking the properties of grout flow, flexural strength, and compressive strength as the evaluation indicator. The specific ratios of ordinary SFPM (O-SFPM), silane coupling agent—SFPM (S-SFPM), cationic emulsified asphalt—SFPM (C-SFPM), and butadiene latex—SFPM (B-SFPM) are shown in Table 8.

The grouting volume of specimens was studied according to the relevant literature [31], with the connected void ratio as the standard. The connected void ratio can affect the actual grouting volume and provide a basis for construction.

(3) $$Q=S\times \bar{H}\times \bar{VV_{c}}\times P_{r}\times (1+a)\times \frac{P_{g}}{1000}$$

where:

*Q* is the quantity of grout used (*g*);

*S* is the perfusion area (mm^2^);

$\bar{H}$ is the average value of pavement thickness (mm);

$\bar{VV_{c}}$ is the average value of the connected void ratio of the matrix asphalt mixture (*%*);

$P_{r}$ is the filling rate (*%*) at the design stage of the fit ratio;

*a* is the grout loss rate, preferably 10.0%;

$P_{a}$ is the density of the grout (g/cm^3^).

Before grouting, the specimen is 63 mm in height and 101.6 mm in diameter, with a connected void ratio of 23.0% and a mass of 961 g. The test results show that the effect of adding the modifier ratio on the density of the grout can be negligible. Thus, the density of the grout is 1.84 g/cm^3^, and the theoretical grouting volume *Q* of the Marshall specimen is 217.97 g according to Equation (3). The main reason is the loss of cement-based grouting material during grouting. To ensure the grouting effect and to control the difference in grouting results caused by the amount of grouting material to the maximum extent, the actual grouting volume is selected as 250 g.

#### 2.2.3. Preparation Process

1. Preparation of cement-based grouting material

The grouting material was prepared according to the optimum water–cement ratio determined. The specific grouting material preparation process is as follows: The solid grouting material was slowly stirred for not less than 1 min at a test temperature of 25 °C using a cement mortar mixer. The slow stirring speed was set to 140 ± 5 r/min for self-rotation and 62 ± 5 r/min for male rotation. After adding the liquid grouting material, the fast stirring was carried out for not less than 4 min, with a speed of 285 ± 10 r/min for self-rotation and 125 ± 10 r/min for male rotation.

2. Preparation of base asphalt mixture

The compaction method and wheel rolling method were used to form the matrix asphalt mixture. In the compaction method, the Marshall compaction machine was used for double-sided compaction 50 times to form the required test pieces. Wheel lapping specimens were prepared by a wheel lapping machine.

3. SFPM preparation

Based on the factors and level design of the SFPM preparation process, grouting was carried out based on the “vibratory infiltration method” [32].

The preparation steps are as follows.

(1) For Marshall specimens, the sides and bottom of the specimens were sealed with plastic tape and cling film after cooling to room temperature and placed vertically at the center of the cement shaker. Rutting specimens were cooled to room temperature without removing the mold and placed at the center of the cement shaker.

(2) Preset vibration frequency and vibration time of the shaking table were determined. The grout was made from the edge of the base asphalt mixture to the center. In the pouring process, it should be poured while vibrating to ensure that the base asphalt mixture is fully filled and dense.

(3) When the surface of the substrate asphalt mixture overflows with cement-based slurry and the grouting material no longer seeps down, close the shaking table and scrape off the excess grouting material on the top surface of the specimen with a rubber scraper until the coarse aggregate leaks out.

(4) The grouted specimens were maintained at a temperature of 20 °C ± 2 °C and relative humidity of >95% for 1 d, 3 d, 7 d, and 28 d.

**Figure 5:**
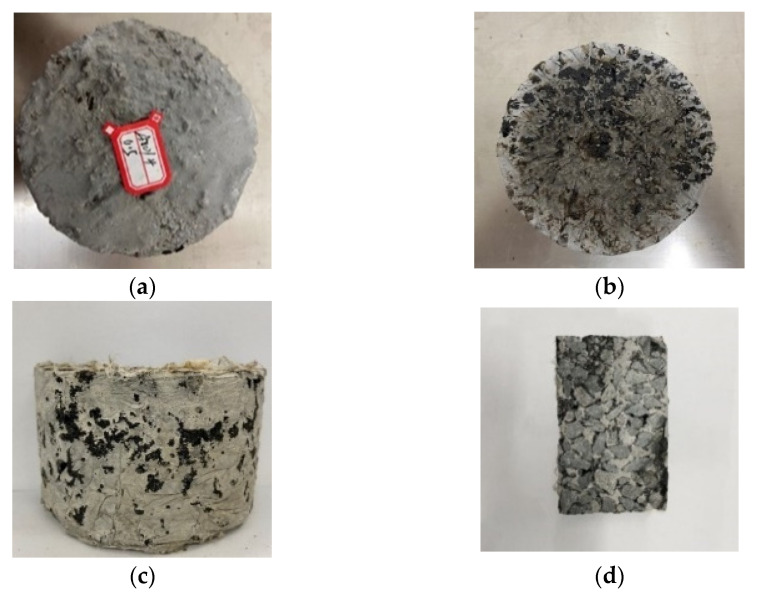
Grouting effect of the specimen: (**a**) Top surface of the specimen; (**b**) Bottom surface of the specimen; (**c**) Side of the specimen; (**d**) Specimen cross section.

The grouting effect of the specimen is shown in Figure 5.

### 2.3. Tests

#### 2.3.1. Performance Test

1. Marshall Stability.

**Figure 6:**
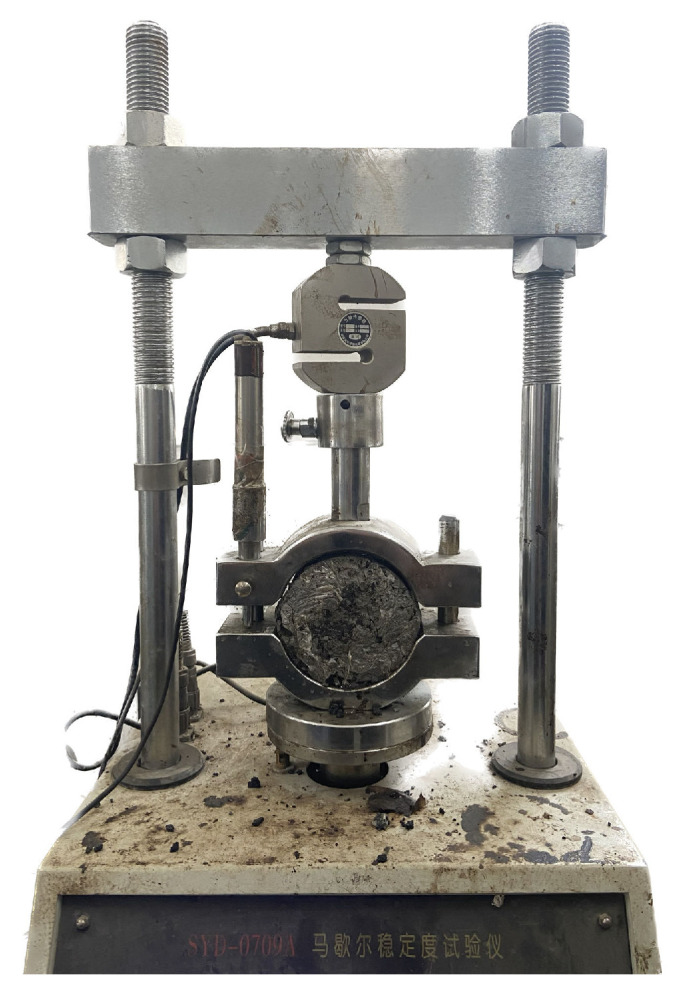
Marshall stability test.

As one of the early design criteria for asphalt pavement, Marshall stability (MS) can reflect the deformation resistance of asphalt mixtures to some extent. To determine the effect of different modifiers on the mechanical properties of semi-flexible pavement mixtures, the ASTM D6927-15 MS test (shown in Figure 6) was used to measure the mechanical properties of semi-flexible pavement mixtures mixed with different modifiers by measuring the MS evaluation.

2. Rutting test.

**Figure 7:**
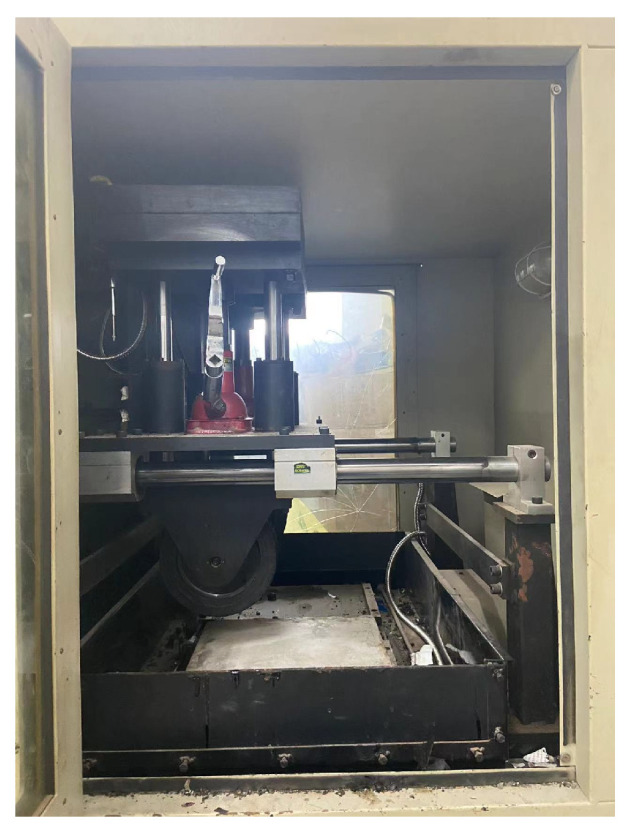
Rutting test.

The high-temperature stability of semi-flexible pavement is usually characterized by dynamic stability (DS), which reflects the high-temperature resistance to rutting. Thus, the AASHTO T324-04 rutting test (shown in Figure 7) was used to evaluate the high-temperature stability of semi-flexible pavement mixtures mixed with different modifiers.

3. Bending test breaking strain at −10 °C.

**Figure 8:**
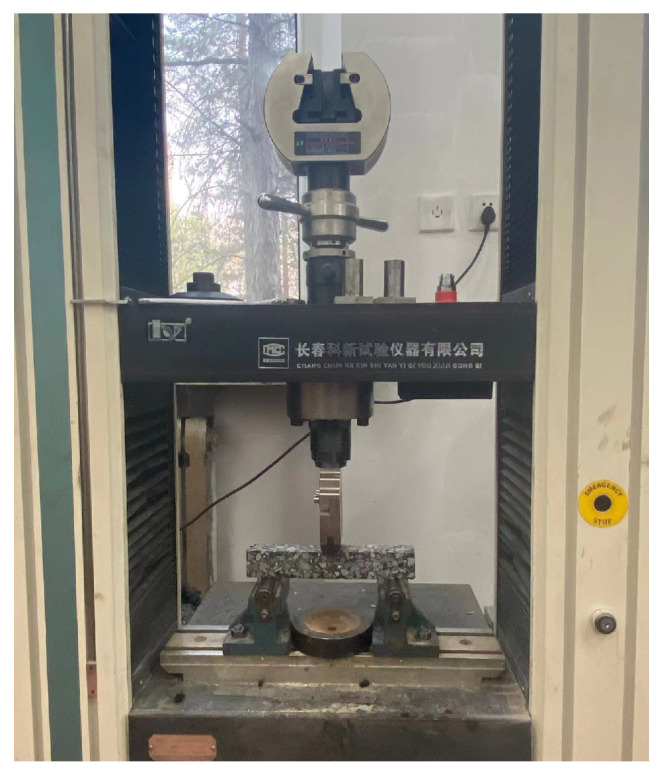
Bending test breaking strain at −10 °C.

For the evaluation of the low-temperature performance of SFPM, the evaluation method of common asphalt mixtures, i.e., the breaking strain bending test at −10 °C, was still used as the low-temperature performance evaluation indicator. Therefore, the AASHTO T283 bending test (shown in Figure 8) was used to test the low-temperature crack resistance of SFPM.

4. Freeze–thaw splitting test.

**Figure 9:**
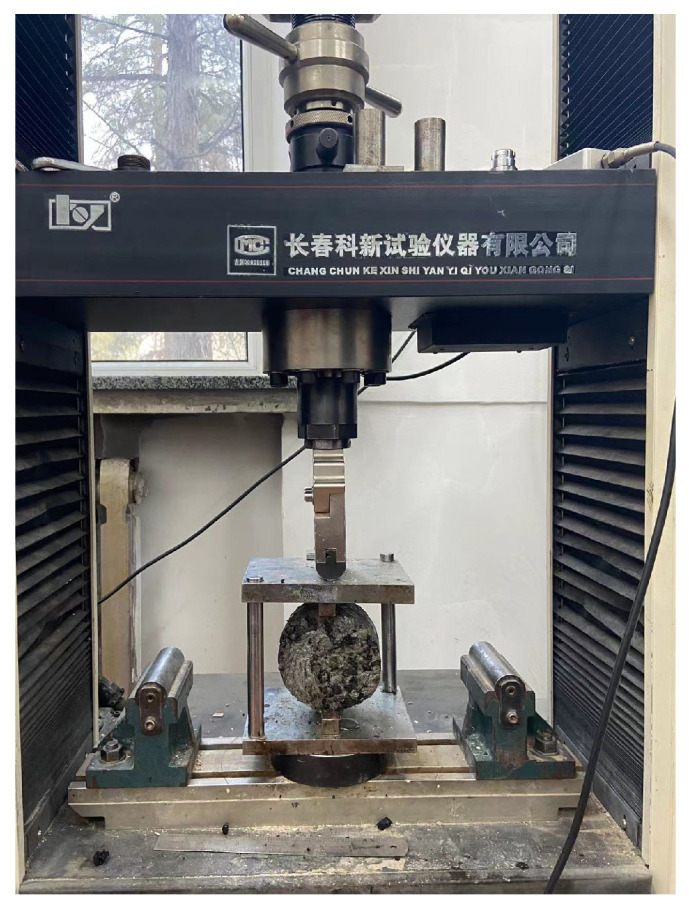
Freeze–thaw splitting test.

The preliminary study showed that the water immersion Marshall test has certain limitations in evaluating the water stability performance of semi-flexible pavement, mainly in residual stability higher than 100%. Thus, the AASHTO T283 freeze–thaw splitting test (shown in Figure 9) was used to determine the water stability of semi-flexible pavement.

#### 2.3.2. Mechanical Test

1. Pull-out test.

**Figure 10:**
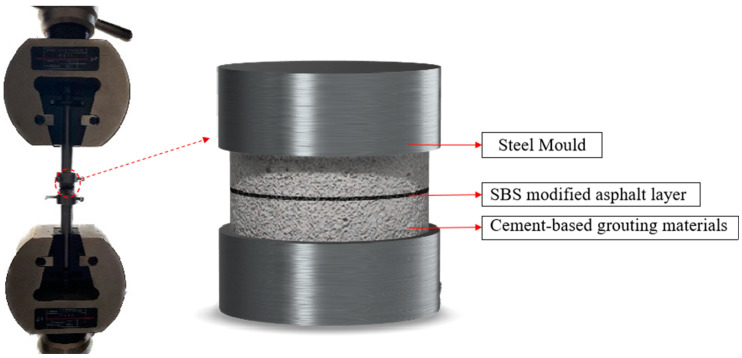
Schematic diagram of the pull-out test.

In this study, a pull-out test was introduced to quantify the effect of the modifier on improving the interfacial strength of the cement–asphalt interface in SFPM. The test is shown in Figure 10. The main experimental steps are as follows.

(1) Sample preparation: The cementitious grouting material was poured into cylindrical specimens with a diameter of 25 mm and a thickness of 15 mm. After 3 d of maintenance, a small amount of SBS asphalt was applied on the surface of the specimens. Two specimens prepared from the same cementitious grouting material were squeezed. The thickness of asphalt film was controlled at 10 (±1) μm.
(2) Sample assembly: the samples prepared in (1) were fixed with two direct stretching jigs after being maintained to a specified age.
(3) Test operation: The moving beam was adjusted to the appropriate position. Then, the direct tensile jig was fixed to the universal testing machine, and tensile damage was performed to the interface with a tensile speed of 10 mm/min. At least three sets of parallel tests were conducted for each interface.

2. SEM and EDS.

In this study, the microscopic morphology of different SFPMs was observed by SEM to investigate the morphological characteristics and the strength formation mechanism of different SFPMs. The chemical elements contained in the SFPM were scanned by EDS. The samples used for SEM testing were maintained for 28 d for observation. Before observation, the dried samples were fixed with conductive resin and coated with a thin layer of gold due to the poor electrical conductivity of SFPM.

## 3. Results and Discussion

### 3.1. Optimal Preparation Process

#### 3.1.1. Orthogonal Experimental Design Method

The orthogonal experimental design method is to study multiple factors and levels. According to orthogonality, part of the representative experiments is selected from all experiments, which are “uniformly dispersed, neat and comparable”. It is one of the commonly used scientific methods to analyze the trend and results of all experiments by designing some experiments with an “orthogonal TABLE” due to the efficient, fast, and economic features [33]. Therefore, the orthogonal experimental design method was used in this study to analyze the preparation process parameters affecting the performance of SFPMs. A four-factor, three-level (3^4^) experimental design was conducted with shaking TABLE vibration frequency, time, modifier type, and dosage as the main factors to obtain the best dosage and preparation process corresponding to the three modifiers.

Taking S-SFPM as an example, the four factors are modifier admixture, concrete curing time, vibration frequency, and vibration time. The vibration frequency of 42 Hz/63.3 Hz for testing was selected according to the vibration frequency of the double-steel-wheel vibratory roller used in the actual project. Three levels of vibration frequency interval of 50–70 Hz were selected in this study, with 10 Hz as an interval. The factors and levels of the experimental design are shown in Table 9. The orthogonal test design TABLE was obtained against the standard orthogonal Table 9 (3^4^), as shown in Table 10.

The MS, DS, flexural–tensile strain (ε), and the freeze–thaw and split residual strength ratio (TSR) of SFPM were selected as the road performance evaluation indicators of S-SFPM. To ensure the credibility of the test results, three sets of parallel specimens were established for each group of tests, and their average values were used as the final experimental results. The specific test results are shown in Table 11.

#### 3.1.2. Gray Correlation Analysis Method

In systems engineering, the gray system refers to the system with part of known information and part of unknown information. On this basis, the gray system theory is mainly applicable to systems with incomplete information, and its scope of application fits well with the orthogonal experimental design method. Thus, it can be used to study the partially known information extracted by the orthogonal experimental design method and predict the overall system situation.

On that basis, the gray correlation analysis method is established by combining the correlation degree, and the statistical analysis of multiple factors is conducted. The sample data of each factor are used to describe the strength, size, and order of the relationship between the factors with the gray correlation degree. The correlation degree between the factors is expressed with the dynamics of factor changes reflected by the sample data.

1. Gray system settings.

The characteristic sequence *X*_0_ of the gray system and the comparison sequence *X_i_* are set as follows:

(4) $$X_{0}=\left[x_{0}(1),\;x_{0}(2),\;x_{0}(3),\;\cdots \cdots x_{0}(n)\right]$$

(5) $$X_{i}=\left[x_{i}(1),\;x_{i}(2),\;x_{i}(3),\;\cdots \cdots x_{i}(n)\right]$$

where $X_{0}(i)$ is the most desirable value among the evaluation indicators.

For the S-SFPM, the selected evaluation indicators, i.e., MS, DS, ε, and TSR, are all positive. The corresponding road performance of the S-SFPM improves with the increase in MS, DS, ε, and TSR. Therefore, test data with higher values were selected as the ideal value, and the gray system was finally obtained. The grey system sequences of the reference sequence *X*_0_ and each comparison sequence *X_i_* are listed in Table 12.

2. Dimensionless processing.


(6) $$X^{*}=\frac{X_{i}-X_{\min}}{X_{\max}-X_{\min}}$$

The different units of measurement lead to different magnitudes and orders of magnitude in the original data, rendering them impracticable for gray correlation analysis. Therefore, before the correlation calculation, the experimental data of each indicator were converted into consistent magnitudes, and Equation (6) was used for polarization. The specific results are shown in Table 13.

3. Correlation coefficient $\xi_{\left(Xi\right)}$ calculation.

The dimensionless parent sequence is $X_{0}$, and the subsequence is $X_{i}$. The correlation coefficient $\xi_{\left(Xi\right)}$ between the comparison series and the reference series at moment *t* was calculated as follows $\xi_{\left(Xi\right)}$

(7) $$\xi_{\left(Xi\right)}=\frac{\Delta_{\min}+\rho \Delta_{\max}}{\Delta_{ot}(t)+\rho \Delta_{\max}}$$

where $\Delta_{ot}(t)$ is the absolute difference between the two sequences at moment *t*, and $\Delta_{ot}(t)=\left|x_{0}(t)-x_{i}(t)\right|$; $\Delta_{\max}$ is the maximum absolute difference between the two sequences at each moment; $\Delta_{\min}$ is the minimum absolute difference between the two sequences at each moment; and ρ is the resolution factor, which is generally between 0 and 1 and set to 0.5 in the calculations.

The experimental data of each indicator after dimensionless processing were calculated according to Equation (7). The absolute difference calculation results are shown in Table 14, and the correlation coefficient calculation results are shown in Table 15.

4. Correlation degree *R_i_* calculation.

Since the correlation degrees of each comparison series and each reference series are reflected by *n* correlation coefficients $\xi_{\left(Xi\right)}$, it is necessary to centralize the correlation information. Thus, the concept of correlation degree was introduced, and the test results of each indicator were once again made more concise and condensed. The correlation degree *R_i_* is the mean correlation coefficient $\xi_{\left(Xi\right)}$ at each moment, which is calculated with Equation (8). It is usually considered that the correlation degree can accurately describe the relative changes among the indicators. The specific calculation results are shown in Table 16.

(8) $$R_{i}=\frac{1}{n}\sum_{i=1}^{n}\xi_{oi}(t)$$

where $\xi_{oi}(t)$ is the correlation coefficient of the parent sequence with the *i* subsequence, and *n* is the number of data in the sequence.

#### 3.1.3. Comprehensive Weighted Scoring Method

As the correlation between the performance indicators derived from the gray correlation analysis can quantitatively reflect the correlation between the factors, it is used as the basis for the comprehensive weighted scoring method of the multi-indicator test so that the data can be evaluated and analyzed holistically.

The comprehensive weighted scoring method refers to the results of the multi-indicator test. The weight was determined according to the importance of each test indicator in the whole test. The test results of multiple indicators were converted into a single indicator of the comprehensive weighted score. Finally, the comprehensive selection of multiple solutions was achieved. Defining $Z_{i}$ as the converted single indicator, i.e., the composite weighted score, and $b_{ij}$ as the weight of the *i*th data in the *j*th indicator, $Z_{i}$ can be calculated as follows:

(9) $$Z_{i}=\sum_{j=1}^{4}b_{ij}\;\times \;\mathrm{Test}\;\mathrm{results}\;\mathrm{of}\;\mathrm{each}\;\mathrm{indicator}$$

where $b_{ij}$ is the proportion of each indicator/the difference between the maximum and minimum of each indicator; i is the number of test groups, set to 1, 2, 3, …, 9; and j is the number of factors, set to 1, 2, 3, and 4.

**Figure 11:**
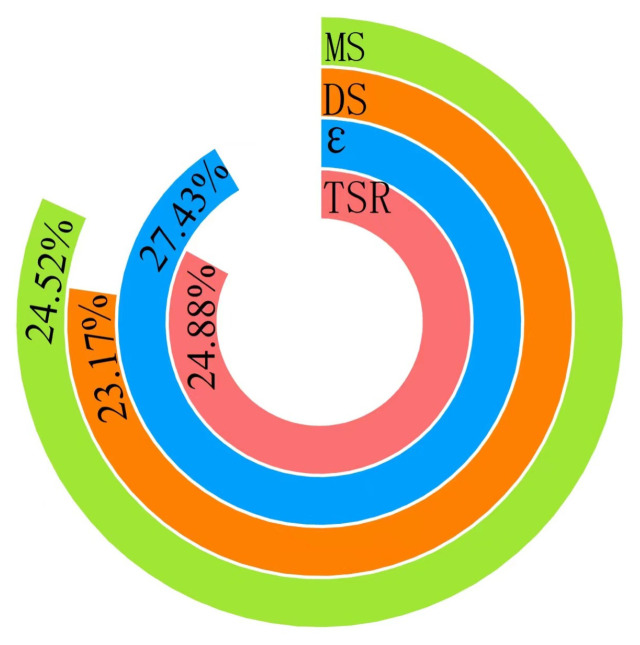
Proportions of each indicator.

The calculated proportions of each indicator are shown in Figure 11, and the comprehensive scoring results are shown in Table 17.

According to the comprehensive scores of each group in Table 17, group 6 has the highest comprehensive score. Thus, group 6 was selected as the best preparation process. According to the orthogonal test design in Table 10, the best doping amount of silane coupling agent is 0.5%, and the best preparation process is 28 d of concrete curing and vibration at the frequency of 50 Hz for 10 min.

Similarly, the best amount of cationic emulsified asphalt is 5%, and the best preparation process is 28 d of maintenance and vibration at the frequency of 60 Hz for 10 min. The best amount of butadiene latex is 10%, and the best preparation process is 28 d of maintenance and vibration at the frequency of 50 Hz for 10 min.

### 3.2. Optimal Modifiers

**Figure 12:**
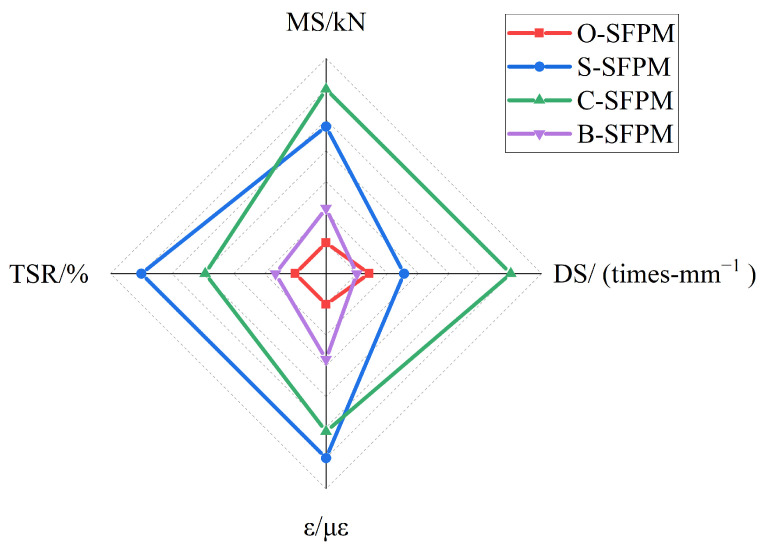
Road performance indicators of different types of SFPMs.

Based on the optimal amount of three modifiers, the road performance of O-SFPMs and three modified SFPMs was compared and analyzed to obtain the best modifier applicable to SFPMs. The road performance indicators of O-SFPMs and three modified SFPMs are shown in Figure 12. Significant differences in the improvement of road performance can be observed between different SFPM modifiers, among which the C-SFPM modified by cationic emulsified asphalt has good performance in terms of high-temperature stability and Marshall strength, while the S-SFPM modified by silane coupling agent has good performance in terms of low-temperature performance and water stability. To further optimize the SFPM preparation process parameters for better overall performance, PCA was introduced to analyze and rank the road performance indicators of the SFPMs with different compositions.

#### 3.2.1. Principal Component Analysis

PCA is a dimensionality-reducing multivariate statistical method that reveals the internal structure among multiple variables through a few principal components, i.e., the effect of selecting the best subset of variables by deriving a few principal components through processing permutations of the original variables and making them retain as much information as possible about the original variables and be uncorrelated with each other as new comprehensive evaluation indicators.

In this study, a variety of SFPMs with different modifiers and O-SFPMs was selected as the evaluation objects, which were recorded as O-SFPM (A1), S-SFPM (A2), C-SFPM (A3), and B-SFPM (A4). Four indicators representing the strength of pavement road performance were selected as the evaluation basis, noted as MS (B1), DS (B2), ε (B3), and TSR (B4).

#### 3.2.2. Applicability Test of Principal Component Analysis

With the help of SPSS 26.0, the raw data were standardized using the Z-score method, and the obtained data were tested for the applicability of PCA using the KMO (Kaiser–Meyer–Olkin) test and Bartlett’s spherical test. In general, the KMO value should be greater than or equal to 0.6, and the sig value should be less than or equal to 0.05. The specific test results are shown in Table 18. The KMO value in this paper is equal to 0.675, indicating a certain correlation between the indicators. The Bartlett’s spherical test result is 230.563, and the sig value is 0.000, indicating that the rejection correlation coefficient is a unit array, i.e., each indicator is correlated. The results of the two tests showed the suitability of the data for PCA.

#### 3.2.3. Principal Component Analysis Process

1. Standardized processing.

Assuming that there are *m* indicator variables for which principal component ($x_{1}$, $x_{2}$, …, $x_{m}$ and *n* objects, and the *j*th indicator of the *i*th evaluation object is $x_{ij}$, the value of each indicator $x_{ij}$ is converted into standardized indicators ${\tilde{x}}_{ij}$ according to Equation (10). The corresponding results of the original and standardized indicators are shown in Table 19.

(10) $${\tilde{x}}_{ij}=\frac{x_{ij}-{\bar{x}}_{j}}{S_{j}}$$

where *i* = 1, 2, 3, 4; *j* = 1, 2, 3, 4; $x_{ij}$ is the *j*th indicator of the *i*th evaluation object; ${\bar{x}}_{j}$ is the sample mean of the *j*th indicator, and ${\bar{x}}_{j}=\frac{1}{n}\sum_{i=1}^{n}x_{ij}$; $S_{j}$ is the sample standard deviation of the *j*th indicator.

Correspondingly, ${\tilde{x}}_{i}=\frac{x_{i}-{\bar{x}}_{i}}{S_{i}}$ (*i* = 1, 2, 3, 4) is defined as the standardized indicator variables.

2. Correlation coefficient matrix R.

The standardized indicators were analyzed to derive the correlation between each indicator using Equations (11) and (12) and build a correlation matrix to understand how the indicators vary relative to the mean, i.e., to identify the correlation between the evaluation indicators. The results are shown in Table 20.

(11) $$R={\left(r_{ij}\right)}_{m\times m}$$

(12) $$r_{ij}=\frac{\sum_{k=1}^{n}{\tilde{x}}_{ki}\cdot {\tilde{x}}_{kj}}{n-1}$$

where *i*, *j* = 1, 2, …, *m*; $r_{ii}=1$, $r_{ij}=r_{ji}$; and rij is the correlation coefficient of the *i*th indicator with the *j*th indicator.

3. Eigenvalues and eigenvectors of the correlation coefficient matrix R.

A principal component is a new variable consisting of a linear combination or mixture of the initial variables. The new variables in this combination (e.g., principal components) are not correlated with each other, and most of the initial variables are compressed into the first component. The results of the principal components obtained using the SPSS 26.0 software are shown in Table 21.

As can be observed from Table 21, the first two principal components explain 99.011% of the total variance, indicating that the two extracted principal components can represent 99.011% of the information of the original name to four strength indicators and are used to evaluate the performance of different modifiers on SFPMs with good credibility. Therefore, the two principal components were extracted as $y_{1}$ and $y_{2}$, and then calculated according to Equation (13). $y_{1}$ and $y_{2}$ are the corresponding principal component coefficients and linear combinations of principal components, respectively.

(13) $$\left\{\begin{array}{l} y_{1}=u_{11}{\tilde{x}}_{1}+u_{21}{\tilde{x}}_{2}+\ldots +u_{n1}{\tilde{x}}_{n}\\ y_{2}=u_{12}{\tilde{x}}_{1}+u_{22}{\tilde{x}}_{2}+\ldots +u_{n2}{\tilde{x}}_{n}\\ \ldots \ldots \\ y_{m}=u_{1m}{\tilde{x}}_{1}+u_{2}{}_{m}{\tilde{x}}_{2}+\ldots +u_{nm}{\tilde{x}}_{n} \end{array}\right.$$

where $\lambda_{1},\lambda_{2},\ldots ,\lambda_{m}$ are the eigenvalues of the correlation coefficient matrix R, and $\lambda_{1}\geq \lambda_{2}\geq \ldots \geq \lambda_{m}\geq 0$; $u_{j}$ are the eigenvectors of the correlation coefficient matrix R, and $u_{j}={\left(u_{1j},u_{2j},\ldots u_{3j}\right)}^{T}$; $y_{1}$ is the first principal component, $y_{2}$ is the second principal component, …, and $y_{m}$ is the *m*th principal component.

Eventually, $y_{1}$ and $y_{2}$ can be calculated. The linear combination is as follows:

$$\left\{\begin{array}{l} y_{1}=0.5401x_{1}+0.4351x_{2}+0.5259x_{3}+0.4923x_{4}\\ y_{2}=0.1736x_{1}+0.7670x_{2}-0.3203x_{3}-0.5271x_{4} \end{array}\right.$$

4. Overall score.

The information contributions ($b_{j}$) of the two principal components and the cumulative contribution rate were calculated with Equations (14)–(16). Thus, the two principal components extracted from the original data were converted into a single indicator, i.e., the composite score, through the contribution rate. The specific results are shown in Table 22.

(14) $$b_{j}=\frac{\lambda_{j}}{\sum_{k=1}^{m}\lambda_{k}}$$

(15) $$a_{p}=\frac{\sum_{k=1}^{p}\lambda_{k}}{\sum_{k=1}^{m}\lambda_{k}}$$

(16) $$a_{p}=\frac{\sum_{k=1}^{p}\lambda_{k}}{\sum_{k=1}^{m}\lambda_{k}}$$

where *j* = 1, 2, …, *m*; $b_{j}$ is the information contribution of the principal component $y_{j}$; $a_{p}$ is the cumulative contribution of principal components $y_{1}$, $y_{2}$, …, $y_{p}$; and *Z* is the composite score.

As can be observed from Table 22, the overall scores of different types of SFPMs rank as follows: C-SFPM > S-SFPM > B-SFPM > O-SFRM. Therefore, adding modifiers improved the road performance of SFPMs to a certain extent, and the C-SFPM had the highest rating of 1.5705. Compared with the rating of the control group of O-SFPM (−1.5756), its improvement effect was more significant. Thus, cationic emulsified asphalt is recommended as the best modifier to improve SFPMs, and the best dose is 5%.

### 3.3. Modification Mechanism Analysis

SFPM is a composite material, with the base asphalt mixture and the cement-based grouting material constituting the aggregate-filler structure together. Thus, the strength of SFPMs is mainly provided by the cement-based grouting material, and the working performance and mechanical properties of the cement-based grouting material directly affect the road performance of SFPMs. In the meantime, when the cement-based grouting material is mixed into the asphalt concrete aggregate, the performance of the semi-flexible pavement is affected by the strength of the cement–asphalt bonding interface. Poor interfacial contact and low interfacial stability lead to insufficient strength of the semi-flexible pavement, which further damages the durability and service life of the pavement. Therefore, this study explored the modification mechanism of cationic emulsified asphalt on SFPMs from two aspects.

#### 3.3.1. Mechanism for Improving the Strength of the Cement–Asphalt Interface

**Figure 13:**
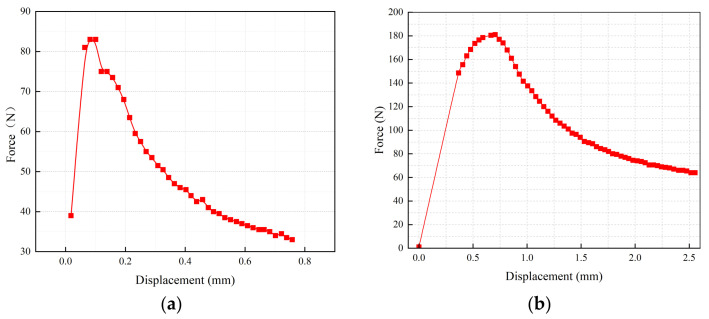
Force–displacement curves of cement–asphalt interfaces: (**a**) Ordinary cement-based grouting material–asphalt; (**b**) Cement-based grouting material–asphalt mixed with 5% cationic emulsified asphalt.

In this study, the force–displacement curves of the cement–asphalt interface of SFPMs before and after modification were obtained by the pull-out test and calculating the interfacial modulus. Thus, the strength of the cement–asphalt interface and the effect of emulsified asphalt on the modification of the cement–asphalt interfacial strength were quantified. The final force–displacement curves under different emulsified asphalt doses are shown in Figure 13.

The corresponding interfacial moduli obtained from the curves are shown in Table 23.

Table 23 shows that the maximum test force and interfacial modulus of the SFPM mixed with 5% cationic emulsified asphalt at the cement–asphalt interface are greatly enhanced compared with the cement–asphalt interface of O-SFPM, which is one of the reasons for the enhanced performance of the SFPM.

#### 3.3.2. Microscopic Morphology and Energy Spectrum Analysis

To further analyze the mechanism by which cationic emulsified asphalt improved the performance of SFPMs, the microstructural characteristics and chemical compositions of SFPMs were analyzed using SEM and EDS energy spectra. After adding the cationic emulsified asphalt, the strength of cement-based grouting materials was affected by cement hydration, emulsified asphalt breaking, etc. The cement hydration products and asphalt intermingled to form a double strength factor action to improve the strength of cement-based grouting materials, and the chemical mechanism of strength formation was analyzed as follows.

(1) The hydration of cement produced Ca(OH)_2_, which reacted with HCl in emulsified asphalt to form CaCl_2_.

Ca(OH)_2_ + 2HCl = CaCl_2_ + 2H_2_O

(2) CaCl_2_ reacted with tricalcium aluminate 3CaO·Al_2_O_3_ in cement to form insoluble complex salts of hydrated calcium chloroaluminate.

9CaCl_2_ + 3CaO·Al_2_O_3_ + 90H_2_O = 3CaO·Al_2_O_3_·3CaCl_2_·30H_2_O

CaCl_2_ + 3Ca(OH)_2_ + 12H_2_O = CaCl_3_·Ca(OH)_2_·12H_2_O

The microstructure and energy spectrum analysis results of C-SFPM are shown in Figure 10.

**Figure 14:**
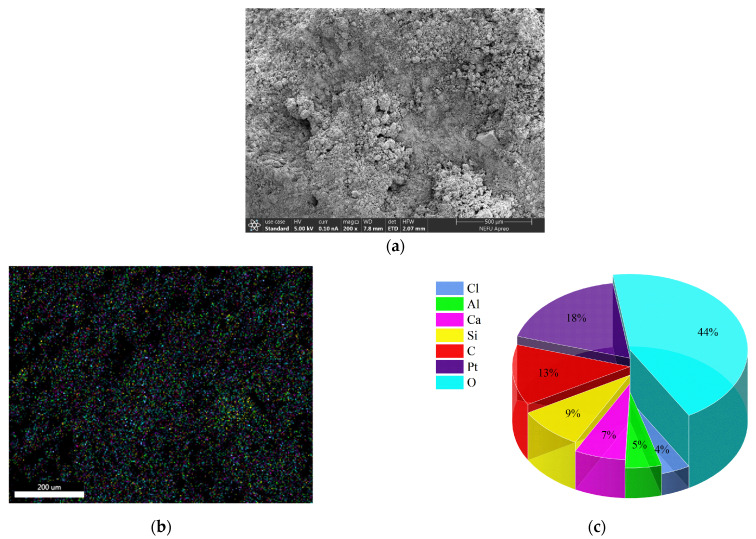

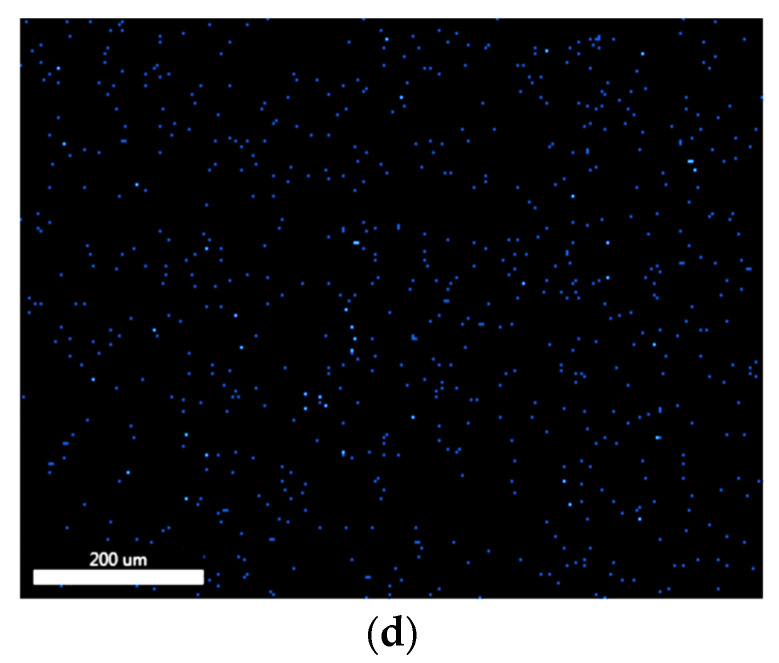
Energy spectra of cationic emulsified asphalt-cement-based grouting material. (**a**) Microstructure of C-SFPM; (**b**) EDS surface scan of C-SFPM; (**c**) Partial element content of C-SFPM; (**d**) Distribution of Cl elements in C-SFPM.

Figure 14b shows the element states of the cationic emulsified asphalt-cement-based grouting material obtained by energy spectra analysis. Although cement does not contain carbon, asphalt contains a large number of carbon elements. However, due to objective factors such as sample preparation, SEM observation is highly susceptible to carbon contamination. Thus, Cl was selected as the characteristic element to express the insoluble complex salt-hydrated calcium chloroaluminate mentioned above and CaCl_2_. Figure 14d shows the distribution of Cl. As can be observed, Cl is evenly distributed in the cementitious material, indicating that the insoluble compound salt-hydrated calcium chloroaluminate is evenly distributed in the cementitious material as its reaction mechanism connects the inorganic and organic materials. It played a certain reactive linkage role and finally formed a kind of inorganic–organic composite structure product in which cement, cement hydration product, and asphalt film penetrated each other, thus improving the strength of cementitious grouting materials. Thus, the performance of SFPMs was improved.

**Figure 15:**
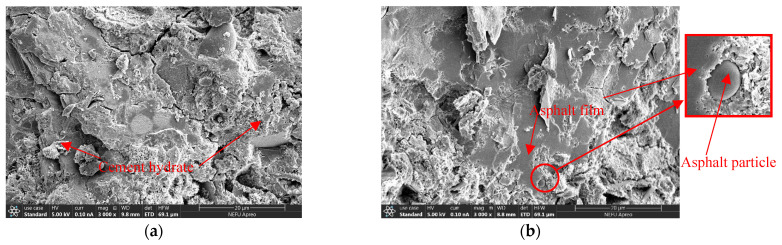
Microstructure diagram (3000×): (**a**) Ordinary cement-based grouting materials; (**b**) Cationic emulsified asphalt-cement-based grouting material.

**Figure 16:**
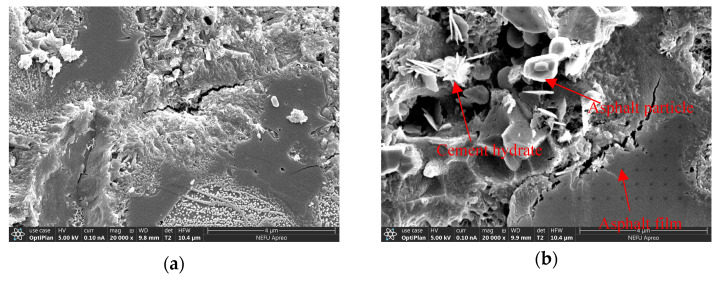
Microstructure diagram (20,000×): (**a**) Ordinary cement-based grouting materials; (**b**) Cationic emulsified asphalt-cement-based grouting material.

**Figure 17:**
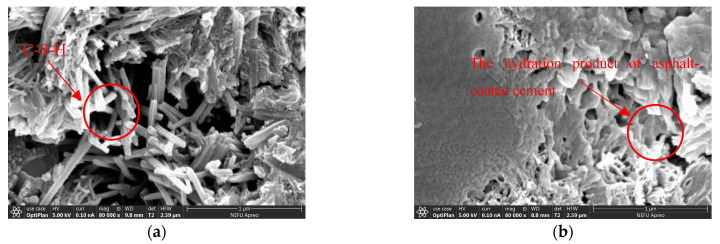
Microstructure diagram (80,000×): (**a**) Ordinary cement-based grouting materials; (**b**) Cationic emulsified asphalt-cement-based grouting material.

The microstructure morphology analysis of the cement-emulsified asphalt composite slurry interface area in cement-based grouting material was carried out, and the results are shown in Figure 15, Figure 16 and Figure 17.

Figure 15a,b show the comparison between the microscopic morphological states of the ordinary cement-based grouting material and the cationic emulsified asphalt-cement-based grouting material magnified 3000 times. The C-SFPM shows smoother parts compared with the O-SFPM, which is attributed to the asphalt film formed after the emulsification of emulsified asphalt. Meanwhile, the air entered during the cement-forming process to form bubble rupture and water evaporation, thus leaving voids in the cement and cement hydration products and making it a porous structure.

Figure 16a,b show the microscopic morphology of ordinary cement-based grouting material and cationic emulsified asphalt-cement-based grouting material with 20,000 times magnification. It can be observed from Figure 16b that the smooth part is the asphalt film formed after the emulsification of emulsified asphalt, and the porous structure is the cement and cement hydration products. Some regular spherical asphalt particles are deformed and attached to the cement hydration products and asphalt film.

Figure 17a,b show the microscopic states of ordinary cement-based grouting material and cationic emulsified asphalt-cement-based grouting material magnified 80,000 times. It can be observed that the internal structure of cementitious grouting materials mixed with cationic emulsified asphalt is different from that of O-SFPMs. As shown in Figure 17a, the internal structure of ordinary cementitious grouting materials has voids and is mainly composed of needle-like and fluffy hydrated calcium silicate (C-S-H). In the meantime, the degree of aggregation is poor compared to that of ordinary cementitious grouting materials, and the structure is looser. As shown in Figure 17b, the internal structure of the cationic emulsified asphalt-cement-based grouting material is a spatial mesh structure formed by cement hydration products, and asphalt is interspersed between the cement hydration products. No obvious interface demarcation line can be observed between asphalt and cement and cement hydration products, thus forming a better interpenetrating network structure with a better aggregation degree and enhanced denseness.

## 4. Conclusions

This study compared and analyzed the effects of different modifiers and their preparation process parameters on SFPM performance improvement and proposed corresponding optimal preparation process parameters. On this basis, the mechanism of SFPM road performance improvement was revealed using SEM and EDS energy spectrum analysis. The main conclusions are as follows.

Firstly, the addition of modifiers significantly changed the roadworthiness of SFPMs, and the modification effects of different modifiers were different. Specifically, the silane coupling agent improved the low-temperature stability of SFPMs and water stability; cationic emulsified asphalt performed well in improving the high-temperature stability and MS. Therefore, the selection of modifiers must consider the actual needs and local climate characteristics.

Secondly, the optimal dosage of silane coupling agent or styrene–butadiene latex was 0.5% and 10%, respectively, for modifying SFPMs. The optimal preparation process parameters were the concrete curing time of 28 d and vibration at the frequency of 50 Hz for 10 min.

Thirdly, compared with the silane coupling agent and butadiene latex, the cationic emulsified asphalt modified SFPM had the best road performance. The recommended preparation process parameters are the cationic emulsified asphalt dose of 5%, the concrete curing time of 28 d, and vibration at the frequency of 60 Hz for 10 min.

Finally, after adding cationic emulsified asphalt, the microstructure of cement-based grouting material changed from the needle-like and fluffy structure with large voids formed by ordinary cement hydration products to the spatial network structure with small voids formed by cement hydration products and asphalt. In the meantime, the strength and stability of the cement–asphalt interface in SFPM were improved. The road performance of SFPM was greatly improved by 242.21%, and the MS, DS, bending and tensile strain, and TSR of C-SFPM increased by 16.72%, 60.16%, 60.55%, and 4.76%, respectively, compared with O-SFPM.

## 5. Recommendation

This study presents a method and framework for enhancing the road performance of SFPM. In the future, low-temperature performance evaluation can be conducted using a range of test methods for a more comprehensive assessment.

## Figures and Tables

**Table 1:** Performance indicators of SBS modified asphalt.

Technical Specifications	Test Results	Specification Requirements	Test Methods
Penetration (25 °C, 0.1 mm)	73.8	60–80	ASTM D5
Softening point (°C)	69.5	≥55	ASTM D36
Ductility (55 °C, cm)	38.6	≥30	ASTM D113

**Table 2:** Main technical indicators of ordinary 42.5 silicate cement.

Technical Specifications	Test Results		Test Methods
Specific surface area	358		ASTM C1329
Standard consistency of cement (%)	27.5		AASHTO T129
Initial setting time (min)	325		AASHTO T131
Final setting time (min)	412		
Flexural strength (MPa)	3 d	5.6	ASTM C78/C78M
	28 d	8.5	
Compressive strength (MPa)	3 d	22.4	ASTM C39/C39M-12a
	28 d	50.3	

**Table 3:** Main technical indicators of the KH-550 silane coupling agent.

Technical Specifications	Technical Requirements	Test Results
Appearance	Transparent liquid	Transparent liquid
Main content	≥97%	99.12
Density	0.9460–0.9560 (20 °C)	0.951
Refractive index	1.4195–1.4205 (25 °C)	1.42
Water dispersibility	Qualified	Qualified

**Table 4:** Cationic emulsified asphalt test results.

Technical Specifications	Technical Requirements	Test Results
Ionic charge		+ ^1^
Residue on 1.18 mm sieve (%)	≤0.10	0.06
Residue content (%)	≥50.0	60.0
Residue solubility (%)	≥97.5	99.4
Residue needle penetration at 25 °C (mm)	60.0–140.0	80.5
Residue ductility at 15 °C (cm)	≥40.0	79.6

^1^ The particle charge “+” represents cationic emulsified asphalt.

**Table 5:** Styrene-butadiene latex test results.

Items	Requirements	Test Results
Appearance	Creamy white liquid with blue light	Creamy white liquid with blue light
Ionic charge	-	-
Residue content (%)	≥50.0	50
pH	6–7	6.4
Viscosity (cps)	1000–1200	1120

**Table 6:** Aggregate gradation.

Type	Passing (%)							
	16.0 mm	13.2 mm	4.75 mm	2.36 mm	0.6 mm	0.3 mm	0.15 mm	0.075 mm
SFAC-13	100	90–100	10–30	5–22	4–15	3–12	3–8	1–6
S-1	100	95	20	10.5	9.5	7.5	5.5	3.5
S-2	100	95	20	13.5	9.5	7.5	5.5	3.5
S-3	100	95	20	16.5	9.5	7.5	5.5	3.5

**Table 7:** Main technical specifications of the matrix asphalt mixture.

Technical Specifications	Test Results	Requirements	Statute
Marshall specimen height (mm)	63	63.5 ± 1.3	ASTM D6927
Marshall stability (kN)	3.39	≥3	
Flow value (0.1 mm)	32.5	20–40	ASTM D1559
Air voids (%)	25.8	20–30	AASHTO T166
Connected air voids (%)	23.0	≥16.0	
Leakage losses (%)	0.5	≤0.8	AASHTO T305
Flying dispersion loss (%)	13.8	≤15	AASHTO T96

**Table 8:** Cement-based grout ratios and technical parameters.

Number	Dosage (%)	Water-to-Ash Ratio	Flow Rate (s)	Compressive Strength (MPa)	Flexural Strength (MPa)
O-SFPM	0	0.50	10.9	50.3	8.5
S-SFPM	0.3	0.50	11.5	47.2	8.9
	0.5	0.50	11.8	45.2	9.1
	0.7	0.50	12.1	43.5	8.2
C-SFPM	5	0.48	9.8	48.7	7.9
	10	0.46	11.2	46.3	7.4
	15	0.44	13.4	42.5	6.7
B-SFPM	5	0.48	11.7	34.2	9.2
	10	0.45	12.8	39.6	9.8
	15	0.43	13.5	37.1	8.9

**Table 9:** Orthogonal test design factors and levels.

Level	Factors			
	Dosage (%)	Concrete Curing Time (d)	Vibration Frequency (Hz)	Vibration Time (min)
1	0.3	3	50	5
2	0.5	7	60	10
3	0.7	28	70	15

**Table 10:** Orthogonal test design.

Test Number	Dosage (%)	Concrete Curing Time (d)	Vibration Frequency (Hz)	Vibration Time (min)
1	0.3	3	50	5
2	0.3	7	60	10
3	0.3	28	70	15
4	0.5	3	60	15
5	0.5	7	70	5
6	0.5	28	50	10
7	0.7	3	70	10
8	0.7	7	50	15
9	0.7	28	60	5

**Table 11:** S-SFPM orthogonal test results.

Test Number	MS (kN)	DS (Times-mm^−1^)	ε (με)	TSR (%)
1	14.96	9864.37	1269.55	79.36
2	19.93	12,607.58	1784.68	87.65
3	21.89	13,758.22	1824.61	86.96
4	20.11	14,087.23	1957.06	87.29
5	19.24	13,355.30	1978.39	89.30
6	22.55	14,328.78	2296.27	92.06
7	19.35	13,569.06	1826.47	85.14
8	21.30	13,726.63	2099.83	89.19
9	20.52	14,568.75	2007.40	92.40

**Table 12:** Grey system sequences.

	MS (kN)	DS (Times-mm^−1^)	ε (με)	TSR (%)
*X*_0_	22.55	14,568.75	2296.27	92.40
*X*_1_	14.96	9864.37	1269.55	79.36
*X*_2_	19.93	12,607.58	1784.68	87.65
*X*_3_	21.89	13,758.22	1824.61	86.96
*X*_4_	20.11	14,087.23	1957.06	87.29
*X*_5_	19.24	13,355.30	1978.39	89.30
*X*_6_	22.55	14,328.78	2296.27	92.06
*X*_7_	19.35	13,569.06	1826.47	85.14
*X*_8_	21.30	13,726.63	2099.83	89.19
*X*_9_	20.52	14,568.75	2007.40	92.40

**Table 13:** Dimensionless processing results.

*X*_0_	1	1	1	1
*X*_1_	0	0	0	0
*X*_2_	0.65	0.58	0.50	0.64
*X*_3_	0.91	0.83	0.54	0.58
*X*_4_	0.68	0.90	0.67	0.61
*X*_5_	0.56	0.74	0.69	0.76
*X*_6_	1.00	0.95	1.00	0.97
*X*_7_	0.58	0.79	0.54	0.44
*X*_8_	0.84	0.82	0.81	0.75
*X*_9_	0.73	1.00	0.72	1.00

**Table 14:** Absolute difference calculation results.

	$\Delta_{o1}$	$\Delta_{o2}$	$\Delta_{o3}$	$\Delta_{o4}$
*X*_1_	1	1	1	1
*X*_2_	0.35	0.42	0.5	0.36
*X*_3_	0.09	0.17	0.46	0.42
*X*_4_	0.32	0.1	0.33	0.39
*X*_5_	0.44	0.26	0.31	0.24
*X*_6_	0	0.05	0	0.03
*X*_7_	0.42	0.21	0.46	0.56
*X*_8_	0.16	0.18	0.19	0.25
*X*_9_	0.27	0	0.28	0

**Table 15:** Correlation coefficient calculation results.

	$\xi_{01}$	$\xi_{02}$	$\xi_{03}$	$\xi_{04}$
*X*_1_	0.33	0.33	0.33	0.33
*X*_2_	0.59	0.54	0.50	0.58
*X*_3_	0.85	0.75	0.52	0.54
*X*_4_	0.61	0.83	0.60	0.56
*X*_5_	0.53	0.66	0.62	0.68
*X*_6_	1.00	0.91	1.00	0.94
*X*_7_	0.54	0.70	0.52	0.47
*X*_8_	0.76	0.74	0.72	0.67
*X*_9_	0.65	1.00	0.64	1.00

**Table 16:** Correlation degree calculation results.

$R_{01}$	$R_{02}$	$R_{03}$	$R_{04}$	$\sum R_{0i}$
0.6512	0.7181	0.6067	0.6419	2.6180

**Table 17:** Overall scoring results.

Test Number	Dosage (%)	Concrete Curing Time (d)	Vibration Frequency (Hz)	Vibration Time (min)	Overall Rating
1	0.3	3	50	5	3023.13
2	0.3	7	60	10	3898.22
3	0.3	28	70	15	4223.41
4	0.5	3	60	15	4143.98
5	0.5	7	70	5	4148.44
6	0.5	28	50	10	4490.61
7	0.7	3	70	10	4170.88
8	0.7	7	50	15	4278.91
9	0.7	28	60	5	4289.08

**Table 18:** Applicability test of principal component analysis.

Inspection Indicator		Results	Inspection Requirements
KMO Sampling suitability quantity		0.675	≥0.600
Bartlett’s sphericity test	Approximate cardinality	230.563	-
	Degree of freedom	45	-
	Significance	0.000	≤0.005

**Table 19:** Standardized indicator results.

	MS		DS		ε		TSR	
	$x_{ij}$	${\tilde{x}}_{ij}$	$x_{ij}$	${\tilde{x}}_{ij}$	$x_{ij}$	${\tilde{x}}_{ij}$	$x_{ij}$	${\tilde{x}}_{ij}$
A_1_	20.03	−1.07165	12,485.24	−0.52581	1535.44	−1.17119	85.12	−0.93848
A_2_	22.55	0.56505	14,328.78	−0.39908	2296.27	0.74265	92.06	1.25263
A_3_	23.36	1.09114	19,996.28	1.49602	2165.20	0.91604	89.18	0.34335
A_4_	20.78	−0.58454	11,826.02	−0.57113	1807.24	−0.48750	86.01	−0.65749

**Table 20:** Correlation coefficient matrix R.

	×1	×2	×3	×4
×1	1.000	0.864	0.924	0.824
×2	0.864	1.000	0.608	0.478
×3	0.924	0.608	1.000	0.955
×4	0.824	0.478	0.955	1.000

**Table 21:** Principal component results.

Total Variance Explained						
Ingredients	Initial Eigenvalue			Extraction of the Sum of Squares of Loads		
	Total	Percentage of Variance	Cumulative (%)	Total	Percentage of Variance	Cumulative (%)
1	3.347	83.671	83.671	3.347	83.671	83.671
2	0.614	15.340	99.011	0.614	15.340	99.011
3	0.040	0.989	100.000			
4	6.705 × 10^−17^	1.676 × 10^−15^	100.000			

**Table 22:** Principal component scores and composite scores.

Types	Principal Component $y_{1}$ Score	Principal Component $y_{2}$ Score	Overall Score Z	Ranking
O-SFRM	−1.9279	0.2446	−1.5756	4
S-SFPM	1.4084	−0.9503	1.0326	2
C-SFPM	1.7097	0.9127	1.5705	1
B-SFPM	−1.1902	−0.2070	−1.0276	3

**Table 23:** Interfacial modulus of SFPMs before and after modification.

Interface Type	Maximum Test Force (N)	Interfacial Modulus (MPa)
General cement-based grouting material–asphalt interface	83.0	120.76
Cementitious grouting material with 5% cationic emulsified asphalt—asphalt interface	181.0	413.25

## Data Availability

Not applicable.
